# A multi-scale layer-resolved spiking network model of resting-state dynamics in macaque visual cortical areas

**DOI:** 10.1371/journal.pcbi.1006359

**Published:** 2018-10-18

**Authors:** Maximilian Schmidt, Rembrandt Bakker, Kelly Shen, Gleb Bezgin, Markus Diesmann, Sacha Jennifer van Albada

**Affiliations:** 1 Laboratory for Neural Coding and Brain Computing, RIKEN Center for Brain Science, Wako-Shi, Saitama, Japan; 2 Institute of Neuroscience and Medicine (INM-6) and Institute for Advanced Simulation (IAS-6) and JARA Institute Brain Structure-Function Relationships (INM-10), Jülich Research Centre, Jülich, Germany; 3 Donders Institute for Brain, Cognition and Behavior, Radboud University Nijmegen, Nijmegen, Netherlands; 4 Rotman Research Institute, Baycrest, Toronto, Ontario, Canada; 5 McConnell Brain Imaging Centre, Montreal Neurological Institute, McGill University, Montreal, Canada; 6 Department of Psychiatry, Psychotherapy and Psychosomatics, Medical Faculty, RWTH Aachen University, Aachen, Germany; 7 Department of Physics, RWTH Aachen University, Aachen, Germany; Université Paris Descartes, Centre National de la Recherche Scientifique, FRANCE

## Abstract

Cortical activity has distinct features across scales, from the spiking statistics of individual cells to global resting-state networks. We here describe the first full-density multi-area spiking network model of cortex, using macaque visual cortex as a test system. The model represents each area by a microcircuit with area-specific architecture and features layer- and population-resolved connectivity between areas. Simulations reveal a structured asynchronous irregular ground state. In a metastable regime, the network reproduces spiking statistics from electrophysiological recordings and cortico-cortical interaction patterns in fMRI functional connectivity under resting-state conditions. Stable inter-area propagation is supported by cortico-cortical synapses that are moderately strong onto excitatory neurons and stronger onto inhibitory neurons. Causal interactions depend on both cortical structure and the dynamical state of populations. Activity propagates mainly in the feedback direction, similar to experimental results associated with visual imagery and sleep. The model unifies local and large-scale accounts of cortex, and clarifies how the detailed connectivity of cortex shapes its dynamics on multiple scales. Based on our simulations, we hypothesize that in the spontaneous condition the brain operates in a metastable regime where cortico-cortical projections target excitatory and inhibitory populations in a balanced manner that produces substantial inter-area interactions while maintaining global stability.

## Author summary

The mammalian cortex fulfills its complex tasks by operating on multiple temporal and spatial scales from single cells to entire areas comprising millions of cells. These multi-scale dynamics are supported by specific network structures at all levels of organization. Since models of cortex hitherto tend to concentrate on a single scale, little is known about how cortical structure shapes the multi-scale dynamics of the network. We here present dynamical simulations of a multi-area network model at neuronal and synaptic resolution with population-specific connectivity based on extensive experimental data which accounts for a wide range of dynamical phenomena. Our model elucidates relationships between local and global scales in cortex and provides a platform for future studies of cortical function.

## Introduction

Cortical activity has distinct but interdependent features on local and global scales, molded by multi-scale connectivity. Data from multiple species including macaque indicate that the ground state of cortex locally features asynchronous irregular spiking with low pairwise correlations [1] and low, layer-specific spike rates [2, 3] with inhibitory rates exceeding excitatory ones [4–6], and activity fluctuations on multiple timescales [7]. Globally, resting-state activity has characteristic patterns of inter-area correlations [8, 9] and propagation [10]. These interactions are layer-specific and distinct between feedback and feedforward directions [11–13]. We present a full-density multi-scale spiking network model in which these features emerge from its detailed structure.

Most cortical models concentrate on either the local or the global scale, using two basic approaches. The first approach represents each neuron explicitly in networks ranging from local microcircuits to small numbers of areas [14, 15]. The second describes large-scale cortical dynamics by simplifying ensemble dynamics to few differential equations. These models predict resting-state oscillations in a metastable regime [16–19] and reproduce the frequency specificity of inter-area interactions [20].

Cortical processing is not restricted to one or few areas, but results from complex multi-area interactions [21, 22]. Simultaneously, dense within-area connectivity [23, 24] suggests the importance of local processing, where the population-specific connectivity underlies multidimensional functional properties [25] and supports a set of computational principles that underlie sensory processing across the cortex [26, 27]. Capturing both aspects requires combining detailed features of local microcircuits with realistic inter-area connectivity. Modeling at cellular resolution enables testing the equivalence with population models instead of assuming it a priori.

Two main obstacles of multi-scale simulations are gradually being overcome. First, recent progress in simulation technology enables the efficient use of supercomputers [28]. Second, systematic connectivity data is increasingly available [29, 30]. However, statistical predictions remain necessary to fully specify large cortical network models. Consequently, few large-scale spiking network models have been simulated to date, and existing ones heavily downscale the number of synapses per neuron [31, 32] (but see [33]), affecting network dynamics [34].

We here investigate a spiking multi-area network model of macaque visual cortex, covering the scales of single neurons, microcircuits, and cortical areas. The connectivity map, derived in [35], customizes that of the microcircuit model of [36] to each area based on its architecture and adds layer-specific inter-area connections. Each area is represented by a 1 mm^2^ microcircuit with the full density of neurons and synapses. A mean-field method [37] refines the connectivity to fulfill the basic dynamical constraint of nonzero and non-saturated activity. By combining simple single-neuron dynamics with complex connectivity, the model enables studying the influence of the connectivity itself on the network dynamics.

We first describe the refinement of the connectivity by dynamical constraints, leading to plausible spike rates. Next, we vary cortico-cortical synaptic strengths and find that with increased coupling, connections onto inhibitory neurons must outbalance connections onto excitatory neurons for stability at low rates. The resulting network state reproduces spiking statistics of V1 resting-state data [38] and yields population bursts reflecting a metastable regime [39–42]. Outside this metastable regime, the spiking statistics deviate considerably from the experimental data. Our findings thus extend previous works demonstrating metastability of cortical networks via modeling [16–19] by unifying microscopic and macroscopic descriptions and supporting the hypothesis that plausible spiking statistics require cortex to be poised in a metastable regime. Analyzing the order of activation of the areas reveals that the population bursts propagate mainly in the feedback direction. Subsequently we show that, for intermediate cortico-cortical synaptic strengths, inter-area correlation patterns resemble fMRI functional connectivity [43]. Finally, we observe directional differences in laminar patterns of inter-area communication that reflect both structural relationships and dynamical states. Our work provides a platform for future studies addressing spiking-level functional properties and for the development of analogous models of other cortical regions. Preliminary results have been presented in abstract form [44].

## Materials and methods

**Fig 1:**
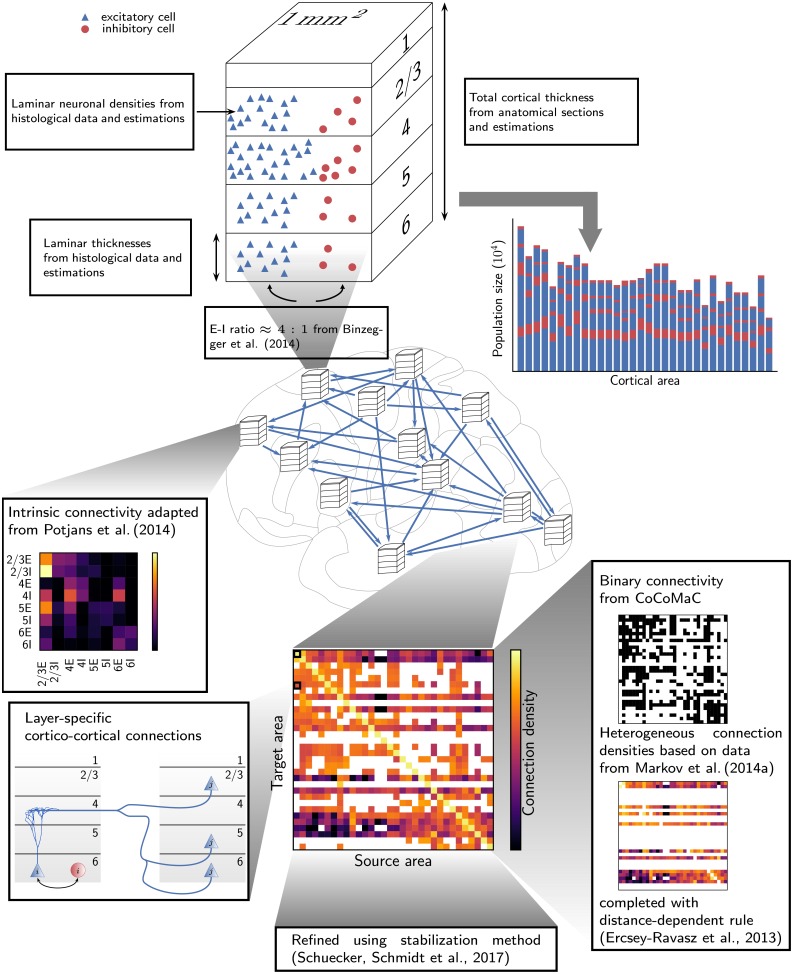
Construction principles of the model. Top: Determination of the population sizes. The size of a population is computed as the product of the layer-specific cell density and its volume computed as thickness times the fixed surface area of 1 mm^2^. Population sizes decrease along the gradient of architectural types. The ratio between excitatory and inhibitory neurons is taken from layer-specific data of [51] leading to an average proportion of ∼80% excitatory and ∼20% inhibitory cells. Figure adapted from [35] and [36] (with permission). Bottom: Construction of the model connectivity. Local connectivity within areas is based on the microcircuit model of [36]. Cortico-cortical connections are first determined on the area level from binary data from the CoCoMac database [29, 61] and quantitative tracing data from [30], which is completed using the exponential fall-off of connection density with inter-area distance [65]. The resulting connectivity spans six orders of magnitude, as shown in the matrix plot of area-averaged indegrees (center bottom). Synapses between each pair of cortical areas are then distributed over source and target layers based on layer-specific tracing data from [66] and CoCoMac. Synapses in the receiving area are subsequently assigned to cells according to layer- and cell-type-specific dendritic densities from [51]. These derivations result in distinct laminar patterns between feedforward, feedback, and lateral connections. Based on a theoretical method using mean-field theory [37], the resulting connectivity matrix is refined to improve the phase space of the network. The detailed derivation is published in [35].

The model comprises 32 areas of macaque cortex involved in visual processing in the parcellation of [45], henceforth referred to as FV91 (Table 1). Each area contains an excitatory and an inhibitory population in each of the layers 2/3, 4, 5 and 6 (L2/3, L4, L5, L6), except area TH, which lacks L4. The model, summarized in Table 2, represents each area by a 1 mm^2^ patch. We here give a brief overview of the construction principles underlying the network definition, and refer to [35] for details of the derivation and an analysis of the network structure. Fig 1 summarizes the construction principles leading to the population sizes and the area-, layer-, and population-specific connectivity map. Table 3 lists the neuron and synapse parameters.

**Table 1:** List of areas in the model, encompassing all vision-related areas of macaque cortex in the parcellation of [45].

Areas in the model		
Lobe	Abbreviation	Brain Region
Occipital	V1	Visual area 1
	V2	Visual area 2
	V3	Visual area 3
	VP	Ventral posterior area
	V3A	Visual area V3A
	V4	Visual area 4
	VOT	Ventral occipitotemporal area
	V4t	V4 transitional area
	MT	Middle temporal area
Temporal	FST	Floor of superior temporal area
	PITd	Posterior inferotemporal (dorsal) area
	PITv	Posterior inferotemporal (ventral) area
	CITd	Central inferotemporal (dorsal) area
	CITv	Central inferotemporal (ventral) area
	AITd	Anterior inferotemporal (dorsal) area
	AITv	Anterior inferotemporal (ventral) area
	STPp	Superior temporal polysensory (posterior) area
	STPa	Superior temporal polysensory (anterior) area
	TF	Parahippocampal area TF
	TH	Parahippocampal area TH
Parietal	MSTd	Medial superior temporal (dorsal) area
	MSTl	Medial superior temporal (lateral) area
	PO	Parieto-occipital area
	PIP	Posterior intraparietal area
	LIP	Lateral intraparietal area
	VIP	Ventral intraparietal area
	MIP	Medial intraparietal area
	MDP	Medial dorsal parietal area
	DP	Dorsal prelunate area
	7a	Area 7a
Frontal	FEF	Frontal eye field
	46	Middle frontal area 46

**Table 2:** Model description after [49].

**Model summary**	
**Populations**	254 populations: 32 areas (Table 1) with eight populations each (area TH: six)
**Topology**	—
**Connectivity**	area- and population-specific but otherwise random
**Neuron model**	leaky integrate-and-fire (LIF), fixed absolute refractory period (voltage clamp)
**Synapse model**	exponential postsynaptic currents
**Plasticity**	—
**Input**	independent homogeneous Poisson spike trains
**Measurements**	spiking activity
**Populations**	
**Type**	Cortex
**Elements**	LIF neurons
**Number of populations**	32 areas with eight populations each (area TH: six), two per layer
**Population size**	*N* (area- and population-specific)
**Connectivity**	
**Type**	source and target neurons drawn randomly with replacement (allowing autapses and multapses) with area- and population-specific connection probabilities
**Weights**	fixed, drawn from normal distribution with mean *J* such that postsynaptic potentials have a mean amplitude of 0.15 mV and standard deviation *δJ* = 0.1*J*; 4E to 2/3E increased by factor 2 [36]; weights of inhibitory connections increased by factor *g*; excitatory weights <0 and inhibitory weights >0 are redrawn; cortico-cortical weights onto excitatory and inhibitory populations increased by factor *χ* and $\chi_{I}\chi$, respectively
**Delays**	fixed, drawn from Gaussian distribution with mean *d* and standard deviation *δd* = 0.5*d*; delays of inhibitory connections factor 2 smaller; delays rounded to the nearest multiple of the simulation step size *h* = 0.1 ms, inter-area delays drawn from a Gaussian distribution with mean *d* = *s*/*v*_t_, with distance *s* and transmission speed *v*_t_ = 3.5 m/s [46]; and standard deviation *δd* = *d*/2, distances determined as the median of the distances between all vertex pairs of the two areas in their surface representation in F99 space, a standard macaque cortical surface included with Caret [47], where the vertex-to-vertex distance is the length of the shortest possible path without crossing the cortical surface [48] (see [35]), delays < 0.1 ms before rounding are redrawn
**Neuron and synapse model**	
**Name**	LIF neuron
**Type**	leaky integrate-and-fire, exponential synaptic current inputs
**Subthreshold dynamics**	$\frac{dV}{dt}=-\frac{V-E_{L}}{\tau_{m}}+\frac{I_{s}\left(t\right)}{C_{m}}$ if (*t* > t* + *τ*_r_) *V*(*t*) = *V*_r_ else $I_{s}\left(t\right)=\sum_{i,k}J_{k}\;e^{-\left(t-t_{i}^{k}\right)/\tau_{s}}\Theta \left(t-t_{i}^{k}\right)$ *k*: neuron index, *i*: spike index
**Spiking**	If *V*(*t*−) < *θ* ∧ *V*(*t*+) ≥ *θ* 1. set t* = *t*, 2. emit spike with time stamp t*
**Input**	
**Type**	Background
**Target**	LIF neurons
**Description**	independent Poisson spikes (for each neuron, fixed rate *ν*_ext_ = *ν*_bg_*k*_ext_ with average external spike rate *ν*_bg_ = 10 spikes/s and number of external inputs per population *k*_ext_, weight *J*)
**Measurements**	
Spiking activity	

**Table 3:** Parameter specification for synapses and neurons.

**Synapse parameters**		
**Name**	**Value**	**Description**
*J* ± *δJ*	Intra-areal connections: 87.8 ± 8.8 pA, cortico-cortical connections scaled as *J*_cc_ = *χJ*, *χ* ∈ [1, 2.5] cortico-cortical connections onto inhibitory populations scaled as $J_{\text{cc}}^{I}=\chi_{I}\chi J$	excitatory synaptic strength
*g*	*g* = 16 in Fig 2A and 2B, *g* = 11 in all other figures	relative inhibitory synaptic strength
*d*_e_ ± *δd*_e_	1.5 ± 0.75 ms	local excitatory transmission delay
*d*_i_ ± *δd*_i_	0.75 ± 0.375 ms	local inhibitory transmission delay
*d* ± *δd*	$d=s/v_{t}\pm \frac{1}{2}s/v_{t}$	inter-area transmission delay, with *s* the distance between areas
*v*_t_	3.5 m/s	transmission speed
**Neuron model**		
**Name**	**Value**	**Description**
*τ*_m_	10 ms	membrane time constant
*τ*_r_	2 ms	absolute refractory period
*τ*_s_	0.5 ms	postsynaptic current time constant
*C*_m_	250 *pF*	membrane capacity
*V*_r_	−65 mV	reset potential
*θ*	−50 mV	fixed firing threshold
*E*_L_	−65 mV	leak potential

Population sizes in the model are derived from a collection of data on laminar and total cortical thicknesses, and area-specific laminar and overall cell densities ([50]; H. Barbas and C. Hilgetag, personal communication). Cell densities in cortex and thus the number of neurons in our model decrease along the visual hierarchy from primary visual cortex V1 (197,936 neurons in total) to area TH (73,251 neurons in total). The ratio between excitatory and inhibitory cells is layer-specific and roughly 4:1 on average; we use values from cat V1 [51] as these underlie the original microcircuit model [36], but the values are close to those from macaque V1 [52]. To estimate missing data on laminar and total thicknesses and neuronal densities, we use a categorization of the areas into architectural types. The architectural type of an area reflects its laminar distinctiveness and the thickness of L4 [53–55].

Based on experimental findings [56, 57], we assume a constant overall synaptic volume density across all areas, which leads to larger numbers of synapses onto neurons in higher areas, in line with an increase in the number of dendritic spines per pyramidal neuron [58–60], due to a hierarchical reduction in neuron density. We distinguish synapses onto a neuron into four distinct types. The first two types are simulated in the model: internal synapses originating within each 1 mm^2^ patch (type I), and cortico-cortical synapses formed by projections from other vision-related cortical areas (type II). Two other types are beyond the scope of the network under investigation: synapses from the same cortical area, but outside the 1 mm^2^ patch (type III), and synapses from other cortical non-visual and non-cortical areas (type IV). For simplicity, inputs from these connections are modeled as spike trains drawn from Poisson processes with stationary rate *ν*_ext_. We make this choice due to the lack of comprehensive data on medium-range connections within areas, such as patchy connectivity; on connections from outside visual cortex; and on the activity levels in resting-state activity of projecting areas. In a future extension of this work, the network would benefit from including such connections or at least diversifying the random inputs.

The connectivity in our model is spatially uniform within each 1 mm^2^ patch. However, to determine the ratio between the numbers of type I and type III synapses we still need a model for distance-dependent connectivity. For this we assume the probability for two neurons to form one or more synapses to decay with the distance between them according to a Gaussian profile. The 1 mm^2^ patch is taken as a disk in the center of a larger disk that represents the full area. The radius of the patch ($R=\sqrt{1/\pi }\;\text{mm}$) determines the cut-off of the Gaussian. The connectivity inside areas is based on the microcircuit model of [36]. The local connectivity of this model is tailored to a cortical area of a given surface area and population sizes of cat V1. We adapt their population-specific connectivity matrix to the compositions of the 32 areas, thereby keeping the proportion of synapses that a neuron in a given population receives from a given other population constant. This translates into requiring that the ratio of indegrees between the connection from population *j* to *i* and from population *l* to *k* is kept constant when translating from the model of [36] (*K*′) to our model for each area *A* (*K*):

$$\begin{array}{c} \frac{K_{ij}\left(A,R\right)}{K_{kl}\left(A,R\right)}=\frac{K_{ij}^{\prime }\left(R\right)}{K_{kl}^{\prime }\left(R\right)}\;\;\;\forall i,j,k,l\;. \end{array}$$

This results in an area-specific conversion factor that scales all the indegrees for a given area. We choose this approach because it approximately preserves a defining characteristic of the local circuit, namely the mean synaptic inputs, which are proportional to the indegrees.

The corticocortical connectivity combines axonal tracing data with statistical regularities to fill in missing values, and is defined in four steps: First, a directed connection between two areas is established if it is included in the CoComac database [29, 45, 61–64] or the retrograde tracing dataset of [30]. Second, the total number of synapses between two areas is based on fractions of labeled neurons (*FLN*) measured in the experiment by [30] or estimated using the exponential fall-off of *FLN* with inter-area distance [65]. Third, we determine laminar patterns based on fractions of supragranular labeled neurons (*SLN*), both measured [66] and estimated using a sigmoidal fit against differences in neuronal densities, and layer-specific data on target [45, 63, 64, 67–72] and source [45, 64, 68, 71, 73, 74] patterns from CoCoMac. Finally, we account for the possibly different laminar positions of synapses and cell bodies in the target areas by computing the conditional probability that a cortico-cortical synapse in a layer *ν* is formed on a dendrite of a specific cell type, using the morphological reconstructions of [51].

Since quantitative area-specific data on non-visual and subcortical inputs are highly incomplete, we use a simple scheme to determine numbers of external inputs: For each area, we compute the total number of external synapses as the difference between the total number of synapses and those from within the 1 mm^2^ patch and other modeled areas, and distribute these such that all neurons in the given area have the same indegree for Poisson sources. In area TH, we compensate for the missing granular layer 4 by increasing the external drive onto populations 2/3E and 5E by 20%. Overall, external inputs amount to approximately 35% of the total inputs to each neuron in the network.

These derivations lead to a connectivity map with similar but non-identical local circuits. The connectivity between areas shows high heterogeneity with connection densities spanning six orders of magnitude, reflecting the similarly high degree of heterogeneity in the quantitative tracing data of [30] (connectivity matrix plot in Fig 1). The laminar patterns of cortico-cortical connections are distinct between feedforward and feedback connections. They mostly follow classical schemes with the important difference that L4 neurons receive a substantial amount of feedback in our network, due to the statistical assignment of synapses to their dendrites in the supragranular layers. As a consequence of the decreasing cell densities combined with a constant synaptic volume density, the average indegrees increase along the visual hierarchy from 3950 synapses per neuron in V1 to 14,000 synapses per neuron in area TH. The overall mean indegree across the entire network is 9500.

The resulting connectivity is modified according to an analytical procedure [37] that integrates fundamental constraints on cortical dynamics into the model while maintaining global stability of the system’s attractors. Details are given in the Results section.

While the number of synapses is population-, layer- and area-specific, we make a simple choice for the synaptic weights to restrict complexity: Individual weights are drawn from Gaussian distributions with mean *J* and width 0.1 *J* for excitatory synapses, and mean −*g J* and width 0.1 *g J* for inhibitory synapses, where *g* is the relative inhibitory synaptic strength (Table 2). There is no comprehensive knowledge about how the strength of cortico-cortical synapses differs from the strength of intra-areal synapses. Therefore, we introduce two factors *χ* and $\chi_{I}$ to scale the weight of synapses onto excitatory neurons by *χ* and onto inhibitory neurons by $\chi_{I}\chi$. For *χ* = 1, we also choose $\chi_{I}=1$, so that cortico-cortical synaptic weights are identical to those of intrinsic synapses. For *χ* > 1, we choose $\chi_{I}=2$, which balances the increased excitation in the system (see Results). Delays are also drawn from Gaussian distributions with fixed mean delays inside areas and distance-dependent cortico-cortical delays (cf. Table 3).

To simulate the network dynamics, we use leaky integrate-and-fire model neurons with exponentially-shaped postsynaptic currents. We choose single-cell parameter values equal to those used by [36] (cf. Table 3). By using a simple neuron model, we limit the complexity of the simulations to bring out the influence of the network connectivity.

### Network simulations

**Fig 2:**
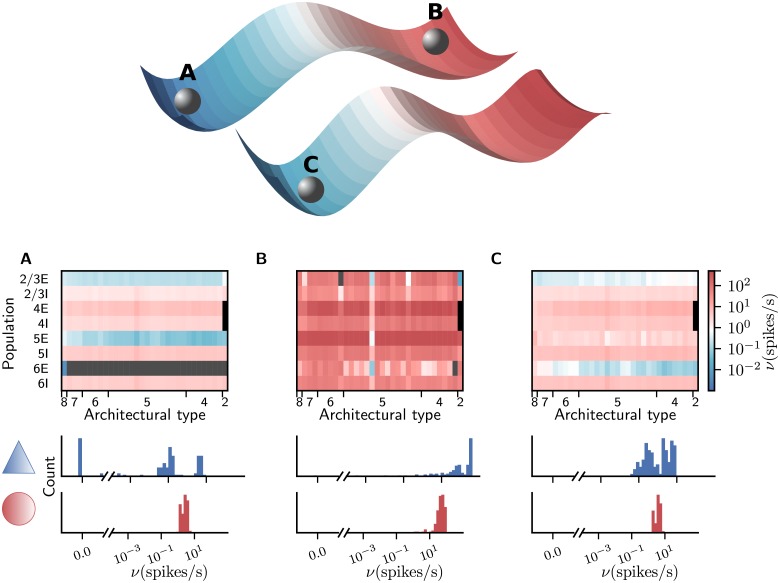
Attractors of the network. Upper part: Qualitative sketch of the phase space of the network with two stable fixed points, with low and unrealistically high activity, respectively. Modifying the connectivity according to the stabilization procedure of [37] shifts the stable low-activity fixed point towards higher rates without decreasing its global stability. The basins of attractions of the two attractors are divided by the separatrix, a sub-manifold containing one or more unstable fixed points. Lower part, upper panels: firing rates encoded in color. Areas are ordered according to their architectural type along the horizontal axis from V1 (type 8) to TH (type 2) and populations are stacked vertically. The two missing populations 4E and 4I of area TH are marked in black and firing rates < 10^-2^ spikes/s in gray. The color scale is identical for all three panels. Lower panels: histograms of population-averaged firing rates for excitatory (blue) and inhibitory (red) populations. The horizontal axis is split into linear- (left) and log-scaled (right) ranges. Simulation parameters: (A) *g* = 16, *ν*_ext_ = 10 spikes/s, *κ* = 1, (B) *g* = 16, *ν*_ext_ = 10 spikes/s, *κ* = 1.125, (C) *g* = 11, *ν*_ext_ = 10 spikes/s, *κ* = 1.125 with the modified connectivity.

We performed simulations on the JUQUEEN supercomputer [75] with NEST version 2.8.0 [76] with optimizations for the use on the supercomputer which were subsequently released in NEST version 2.12.0 [77]. The simulations use 1024 compute nodes (corresponding to 1 rack of JUQUEEN) with 1 MPI process per node and 64 threads per MPI process. A model instance requires about 2GB of working memory on each compute node and takes about 5 minutes for the creation of the network and approximately 12 minutes per 1 s biological time for propagation of the dynamical state. All simulations use a time step of 0.1 ms and exact integration for the subthreshold dynamics of the leaky integrate-and-fire neuron model [78]. Simulations are run for 100.5 s (*χ* = 1.9), 50.5 s (*χ* ∈ [1.8, 2.0, 2.1]), and 10.5 s (*χ* ∈ [1., 1.4, 1.5, 1.6, 1.7, 1.75, 1.8, 2.5]) biological time discarding the first 500 ms. Spike times of all neurons are recorded, except for the simulations shown in Fig 2A and 2B, where 1000 neurons per population are recorded. The digitized workflow reproducing all results and figures of this work was created in compliance with [79] and is available as Python code from https://github.com/INM-6/multi-area-model. The simulation data presented in this manuscript is available from https://web.gin.g-node.org/maximilian.schmidt/multi-area-model-data.

### Analysis methods

**Fig 3:**
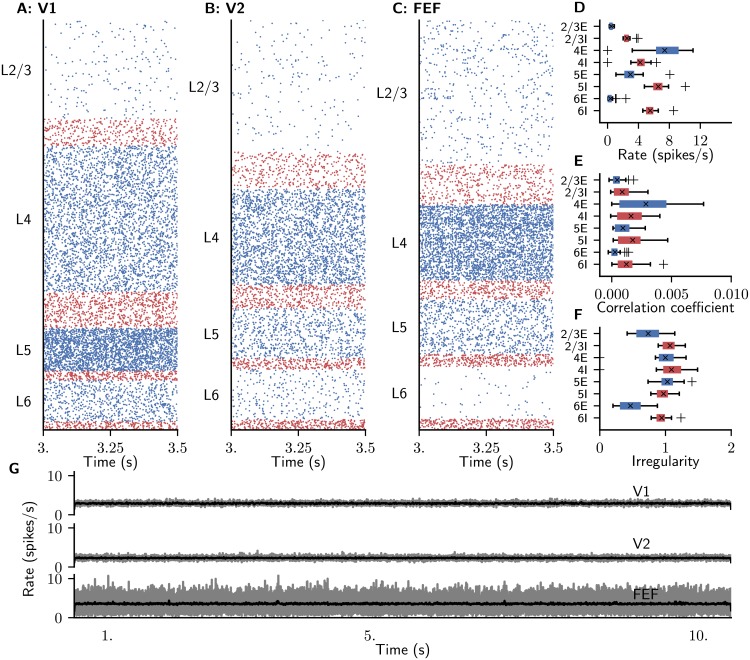
Ground state of the model. (A-C) Raster plot of spiking activity of 3% of the neurons in area V1 (A), V2 (B), and FEF (C). Blue: excitatory neurons, red: inhibitory neurons. (D-F) Spiking statistics across all 32 areas for the respective populations shown as area-averaged box plots. Crosses: medians, boxes: interquartile range (IQR), whiskers extend to the most extreme observations within 1.5×IQR beyond the IQR. (D) Population-averaged firing rates. (E) Average pairwise correlation coefficients of spiking activity. (F) Irregularity measured by revised local variation *LvR* [82] averaged across neurons. (G) Area-averaged firing rates, shown as raw binned spike histograms with 1 ms bin width (gray) and convolved histograms, with a Gaussian kernel (black) of optimal width [80].

Instantaneous firing rates are determined as spike histograms with bin width 1 ms averaged over the entire population or area. In Figs 3G and 5G we convolve the histograms with Gaussian kernels of optimal width using the method of [80], implemented in the Elephant package [81]. Spike-train irregularity is quantified for each population by the revised local variation *LvR* [82] averaged over a subsample of 2000 neurons. The cross-correlation coefficient is computed with bin width 1 ms on single-cell spike histograms of a subsample of 2000 neurons per population with at least one emitted spike per neuron. Both measures are computed on the entire population if it contains fewer than 2000 neurons. To compare the simulated with the experimental power spectrum in Fig 6K, we use the simulated spiking data from 140 neurons (equal to the number of neurons identified in the experimental data), distributed across populations in V1 in proportion to the population sizes. We compute the power spectrogram and power spectral densities using Welch’s method (signal.welch of the Python SciPy library [83] with a ‘boxcar’ window, segment length of 1024 data points and 1000 overlapping points between segments). To make our results as comparable as possible with [38], we follow these authors and disregard neurons with an average spiking rate < 0.56 spikes/s.

We employ analytical mean-field theory to predict the stationary population-averaged firing rates of the model. In the diffusion approximation, which is valid for large numbers of sufficiently independent inputs with small synaptic weights, the dynamics of the membrane potential *V* and synaptic current *I*_s_ of the leaky integrate-and-fire model neurons used in our model are described by [84]

$$\begin{array}{ccc} \tau_{\text{m}}\frac{dV}{dt} & = & -V+I_{s}\left(t\right)\\ \tau_{s}\frac{dI_{s}}{dt} & = & -I_{s}+\mu +\sigma \sqrt{\tau_{\text{m}}}\xi \left(t\right), \end{array}$$

where the input spike trains are replaced by a current fluctuating around the mean *μ* with variance *σ* with fluctuations drawn from a random Gaussian process *ξ*(*t*) with 〈*ξ*(*t*)〉 = 0 and 〈*ξ*(*t*)*ξ*(*t*′)〉 = *δ*(*t* − *t*′). Going from the single-neuron level to a description at the population level, we define the population-averaged firing rate *ν*_*i*_ due to the population-specific input *μ*_*i*_, *σ*_*i*_. The stationary firing rates *ν*_*i*_ are then given by [84]

$$\begin{array}{ll} \frac{1}{\nu_{i}} & =\tau_{\text{r}}+\tau_{\text{m}}\sqrt{\pi }\int_{\frac{V_{\text{r}}-\mu_{i}}{\sigma_{i}}+\gamma \sqrt{\frac{\tau_{\text{s}}}{\tau_{\text{m}}}}}^{\frac{\Theta -\mu_{i}}{\sigma_{i}}+\gamma \sqrt{\frac{\tau_{\text{s}}}{\tau_{\text{m}}}}}e^{x^{2}}\left(1+\text{erf}\left(x\right)\right)\text{d}x\\ & ≕1/\Phi_{i}\left(\nu \right)\\ \mu_{i} & =\tau_{\text{m}}\sum_{j}K_{ij}J_{ij}\nu_{j}+\tau_{\text{m}}K_{\text{ext}}J_{\text{ext}}\nu_{\text{ext}}\\ \sigma_{i}^{2} & =\tau_{\text{m}}\sum_{j}K_{ij}J_{ij}^{2}\nu_{j}+\tau_{\text{m}}K_{\text{ext}}J_{\text{ext}}^{2}\nu_{\text{ext}}, \end{array}$$ (1)

which holds up to linear order in $\sqrt{\tau_{s}/\tau_{\text{m}}}$ and where $\gamma =\left|\zeta \left(1/2\right)\right|/\sqrt{2},$ with *ζ* denoting the Riemann zeta function [85]. We solve this equation for our high-dimensional network by finding the fixed points of the first-order differential equation [86]

$$\begin{array}{c} \dot{\nu }≔\frac{d\nu }{ds}=\Phi \left(\nu \right)-\nu \;, \end{array}$$ (2)

for different initial conditions ***ν***_0_ using the continuous-time dynamics framework of NEST [87], which uses the exponential Euler algorithm, with step size *h* = 0.1, where *s* denotes a dimensionless pseudo-time. To investigate the local stability of the fixed point, we study the evolution of a small perturbation ***δν*** around the fixed point ν* to linear order,

$$\begin{array}{cc} \nu_{i} & =\nu_{i}^{*}+\delta {\tilde{\nu }}_{i}\\ & =\Phi_{i}\left(\nu^{*}+\delta \nu \right)=\Phi_{i}\left(\nu^{*}\right)+\frac{d\Phi_{i}}{d\nu }\delta \nu \\ & =\nu_{i}^{*}+\frac{\partial \Phi_{i}}{\partial \mu_{i}}\sum_{j}\frac{d\mu_{i}}{d\nu_{j}}\delta \nu_{j}+\frac{\partial \Phi }{\partial \sigma_{i}^{2}}\sum_{j}\frac{d\sigma_{i}^{2}}{d\nu_{j}}\delta \nu_{j}\\ & =\nu_{i}^{*}+\sum_{j}G_{ij}\delta \nu_{j}\\ \Rightarrow \delta {\tilde{\nu }}_{i} & =\sum_{j}G_{ij}\delta \nu_{j}\\ \delta \tilde{\nu } & =G\delta \nu \;. \end{array}$$ (3)

The perturbation decays to zero if the maximal real value of the eigenvalues of the effective connectivity matrix ***G***, the Jacobian of **Φ**, is smaller than 1.

To investigate inter-area propagation, we determine the temporal order of spiking based on the location of the extremum of the correlation function for each pair of areas. This measure is chosen to characterize the relative timing of activity fluctuations across areas, as opposed to measures of causal interactions like Granger causality and the directed transfer function [88]. In an analysis of the lag structure of resting-state fMRI, Mitra et al. [10] similarly characterize temporal order using the time-delay matrix derived from the lagged cross-covariance functions. Our method also resembles the assessment of propagation using the relative timing of slow waves in EEG and LFP recordings in different areas [89–91]. In analogy to structural hierarchies based on pairwise connection patterns [92, 93], we look for a temporal hierarchy that best reflects the order of activations for all pairs of areas. This overall characterization of temporal order extracts the essence of the more complex picture provided by the pairwise delays. The hierarchy is based on the cross-covariance function computed between area-averaged firing rates and subsequently convolved with Gaussian kernels with *σ* = 2 ms to obtain smoother curves. We use a wavelet-smoothing algorithm (signal.find_peaks_cwt of the Python SciPy library [83] with peak width Δ = 5 ms) to detect extrema for *τ* ∈ [−100, 100] and take the location of the extremum with the largest absolute value as the time lag. To order areas hierarchically, we determine the peak locations *τ*_*AB*_ of the cross-correlation function for each pair of areas *A*, *B*. We then define a function for the deviation between the distance of hierarchical levels *h*(*A*), *h*(*B*) and peak locations,

$$\begin{array}{c} f\left(A,B\right)=h\left(A\right)-h\left(B\right)-\tau_{AB}\;. \end{array}$$

To determine the hierarchical levels, we minimize the sum of *f*(*A*, *B*) over all pairs of areas,

$$\begin{array}{c} S=\sum_{A,B}f\left(A,B\right)\;, \end{array}$$

using the optimize.minimize function of the scipy library [83] with random initial hierarchical levels. We verified that the initial choice of hierarchical levels does not influence the final result. We obtain hierarchical levels on an arbitrary scale, which we normalize to values *h*(*A*) ∈ [0, 1]∀*A*.

In the context of our spiking network model we define functional connectivity (FC) as the zero-time lag cross-correlation coefficient of the area-averaged synaptic inputs, which we approximate as

$$\begin{array}{c} I_{A}\left(t\right)=\frac{1}{N_{A}}\sum_{i\in A}N_{i}|I_{i}\left(t\right)|=\frac{1}{N_{A}}\sum_{i\in A}N_{i}\sum_{j}K_{ij}|J_{ij}|(\nu_{j}*PSC_{j})\left(t\right)\;, \end{array}$$

with the normalized post-synaptic current *PSC*_*j*_(*t*) = exp[−*t*/*τ*_s_], * indicating convolution, synaptic time constant *τ*_s_, the population firing rate *ν*_*j*_ of source population *j*, mean indegree *K*_*ij*_, and mean synaptic weight *J*_*ij*_ of the connection from *j* to target population *i* containing *N*_*i*_ neurons. The population firing rate *ν*_*j*_ is defined as the spike histogram with bin width 1 ms averaged over the entire population, thus time *t* is in discrete increments of 1 ms.

We compute a BOLD signal from the simulated area-averaged synaptic inputs using the Balloon model [94], implemented in the neuRosim [95] package of R. Synaptic inputs *I*_*A*_(*t*) drive the responses of cerebral blood flow (CBF) *f*(*t*) and cerebral metabolic rate of oxygen (CMRO_2_) *m*(*t*) by linear convolutions

$$\begin{array}{ccc} f(t) & = & 1+\left(f_{1}-1\right)h\left(t-\delta t\right)*I_{A}\left(t\right)\\ m(t) & = & 1+\left(m_{1}-1\right)h\left(t\right)*I_{A}\left(t\right)\\ \text{with}\;h(t) & = & \frac{1}{k\tau_{h}\left(k-1\right)!}{\left(\frac{t}{\tau_{h}}\right)}^{k}e^{-t/\tau_{h}}\;\\ \tau_{f}=4\;s\;, & \tau_{h}=0.242\;\tau_{f}, & \;f_{1}=1.5,\;\left(m_{1}-1\right)=\left(f_{1}-1\right)/2,\;\delta t=1\;s. \end{array}$$

These responses then feed into the Balloon model which is characterized by two dynamical variables *q*(*t*), *v*(*t*):

$$\begin{array}{ccc} \frac{dq}{dt} & = & \frac{1}{\tau_{\text{MTT}}}[f\left(t\right)\frac{E(t)}{E_{0}}-\frac{q(t)}{v(t)}f_{\text{out}}\left(v,t\right)]\\ \frac{dv}{dt} & = & \frac{1}{\tau_{\text{MTT}}}[f\left(t\right)-f_{\text{out}}\left(v,t\right)]\\ \text{with}\;f_{\text{out}}\left(v,t\right) & = & v^{1/\alpha }+\tau \frac{dv}{dt}\\ E(t) & = & E_{0}\frac{m(t)}{f(t)} \end{array}$$

with *τ*_MTT_ = 3 s, *τ* = 10 s, *α* = 0.4, *E*_0_ = 0.4. These two variables determine the relative change of the BOLD signal *S*:

$$\begin{array}{c} \frac{\Delta S}{S}=V_{0}[a_{1}\left(1-q\right)-a_{2}\left(1-v\right)] \end{array}$$

with *a*_1_ = 3.4, *a*_2_ = 1*V*_0_ = 0.03. The parameters are chosen as in [94].

Clusters in the FC matrices are detected by optimizing the modularity of the weighted, undirected FC graph [96]. We use the function modularity_louvain_und_sign of the Brain Connectivity Toolbox (BCT; http://www.brain-connectivity-toolbox.net) with the Q* option, which weights positive weights more strongly than negative weights, as introduced by [97]. The clustering of the structural connectivity is performed with the map equation method [98], which can handle directed connections but no negative weights. In this clustering algorithm, an agent performs random walks between graph nodes according to their degree of connectivity and a certain probability of jumping to a random network node. We choose the probability for a certain target node to be selected to be proportional to the outdegree of the connection, and *p* = 0.15 as the probability of a random jump. The algorithm detects clusters in the graph by minimizing the length of a binary description of the network using a Huffman code.

To investigate causal relations in the network, we compute the conditional Granger causality [99] between pairs of populations. To reduce computational load, we restrict the set of source populations for each target population *i* to those that form a connection with on average more than 1 synapse per target neuron. A vector autoregressive model (VAR) describes the target firing rate *ν*_*i*_(*t*) based on the firing rates of other populations with a maximal time lag of 25 ms corresponding to the rounded maximal delay between any two areas in the network. For each source population *j*, we perform two fits: one using the set of all source populations, yielding *VAR*_{*j*′}_, and one using all source populations except *j*, yielding *VAR*_{*j*′|*j*′ ≠ *j*}_. To determine the causal influence *j* → *i*, we test whether the residual variances of the two VARs are significantly different using Levene’s test [100], which is more robust against non-normally distributed residuals than the F-test.

To study dominant paths in the network, we construct the weighted and directed gain matrix ***G*** with $G_{ij}=K_{ij}\left|J_{ij}\right|\frac{d\mu_{i}}{d\nu_{j}}+K_{ij}J_{ij}^{2}\frac{d\sigma_{i}^{2}}{d\nu_{j}}$ of the network at the population level, where we evaluate the terms $\frac{d\mu_{i}}{d\nu_{j}},\;\frac{d\sigma_{i}^{2}}{d\nu_{ij}}$ at the simulated population-averaged firing rates of the model with *χ* = 1.9. We denote the eigenvalues of ***G*** by λ and define λ_max_ as the eigenvalue with the largest real part. To reflect the near-criticality of the brain, we perform an element-wise division by the real part of λ_max_: ***G***′ = ***G***/[Re(λ_max_)], so that the maximal real part of the eigenvalues λ′ of the resulting matrix ***G***′ is max[Re(λ′)] = 1. This scaling modulates the relative strengths of direct and indirect paths: a larger value of max[Re(λ′)] increases the relative weighting of indirect paths. Subsequently we use the same method as [35] and denote the weight of the edge from population *j* to *i* as $g_{ij}^{\prime }$. The logarithm of the reciprocal of the weight, *d*_*ij*_ = log(1/*w*_*ij*_), defines the distance between two nodes in the graph so that summing the distances corresponds to a multiplication of the corresponding weights. Next, the Bellman-Ford algorithm [101–103] finds the shortest paths between any two nodes of the graph. This algorithm determines the shortest paths emanating from vertex *i* on a graph with *N* vertices in an iterative manner: it initially assigns an infinite path length to all other nodes *k* of the graph. Then, the algorithm loops through all edges (*j*, *k*) of the graph, tests if the path length *p*_*ij*_ plus the distance of the edge *d*_*jk*_ is smaller than the currently stored path length *p*_*ik*_, and, if so, assigns *p*_*ik*_ ← *p*_*ij*_ + *d*_*jk*_. By repeating the loop over all edges *N* − 1 times, the algorithm considers paths of increasing length on every iteration and ultimately uncovers the shortest paths between each pair of vertices. In contrast to Dijkstra’s algorithm Bellman-Ford copes with edges with negative distance values.

### Recording of spiking data from [38]

The experimental recordings are described in [38] and are publicly available [104]. The data consist of sorted spike trains from a 64-electrode array implanted into primary visual area V1 of a lightly anesthetized macaque monkey. The array has 8 electrodes, called shanks, with 8 contacts sites per shank, spanning 1.4 × 1.4 mm horizontally and in depth at 200 *μ*m spacing, covering all cortical layers. For the analysis in Fig 6, we used the 15 minutes of spontaneous activity, where no visual stimulation was provided to the animal. To obtain single-neuron spike trains, [38] performed super-paramagnetic clustering [105] on the high-frequency component (400–5000 Hz) of the recorded signal. Details of the experimental procedures are given in [38]. In our analysis, we distinguish between low-fluctuation and high-fluctuation phases, with low vs. high activity in the frequency range up to 40 Hz. We defined these phases from the power spectrum |*C*(*ω*)|^2^ of the spike histogram for all neurons combined at subsequent intervals of 10 s duration and assign the interval to the low-fluctuation phase if $\int_{0\;\text{Hz}}^{40\;\text{Hz}}{\left|C\left(\omega \right)\right|}^{2}d\omega \leq \theta$, with an empirically determined threshold *θ* = 0.8 ⋅ 10^8^. This leads to 77 intervals being classified as low-fluctuation and 15 intervals as high-fluctuation.

### Macaque resting-state fMRI

Data were acquired from six male macaque monkeys (4 *Macaca mulatta* and 2 *Macaca fascicularis*). All experimental protocols were approved by the Animal Use Subcommittee of the University of Western Ontario Council on Animal Care and in accordance with the guidelines of the Canadian Council on Animal Care. Data acquisition, image preprocessing and a subset of subjects (5 of 6) were previously described [106]. Briefly, 10 5-min resting-state fMRI scans (TR: 2 s; voxel size: 1 mm isotropic) were acquired from each subject under light anesthesia (1.5% isoflurane). Nuisance variables (six motion parameters as well as the global white matter and CSF signals) were regressed using the AFNI software package (afni.nimh.nih.gov/afni). The global mean signal was not regressed.

The FV91 parcellation was drawn on the F99 macaque standard cortical surface template [47] and transformed to volumetric space with a 2 mm extrusion using the Caret software package (http://www.nitrc.org/projects/caret). The parcellation was applied to the fMRI data and functional connectivity computed as the Pearson correlation coefficients between probabilistically weighted ROI timeseries for each scan [43]. Correlation coefficients were Fisher z-transformed and correlation matrices were averaged within animals and then across animals before transforming back to Pearson coefficients.

## Results

### Refinement of connectivity by dynamical constraints

From a dynamical systems perspective, we define a state of the network as a set of mean firing rates for all populations. An attractor is a state towards which the network tends to evolve for many different initial conditions. Since the network receives stochastic external input, individual neurons fluctuate around their mean firing rate. An attractor is locally stable if all eigenvalues of the effective connectivity matrix, defined as the Jacobian of the population-level transfer function obtained from mean-field theory (Eq (1)), have real values < 1. The global stability of an attractor is assessed by the size of its basin of attraction in phase space. This volume is measured by discretizing the phase space into a grid of initial conditions and defining the global stability of an attractor *A* as the proportion of initial conditions leading the system to evolve to *A*. An analysis based on mean-field theory [37] and simulations reveals that across a wide range of configurations of the external input rate *ν*_ext_ and the relative inhibitory synaptic strength *g*, the network possesses a bistable activity landscape with two coexisting locally stable fixed points (Fig 2). In view of the high dimensionality of the system with 254 populations, the bistability in the mean-field theory is found numerically from a pseudo-time integration that yields the stable fixed points [37], in which the set of firing rates for the full set of populations consistently converges to one of two possible states for each combination of *ν*_ext_ and *g*. The simulation results are qualitatively consistent with these mean-field results. We identify the stable fixed points based on the fact that, after a short initial simulation phase (typically ∼100 ms) and regardless of the initial condition, the network settles in either of these states. The first attractor exhibits asynchronous, irregular activity at moderate firing rates except for populations 5E and 6E, which are nearly silent (Fig 2A), while the second features highly synchronized and regular firing with excessive rates (Fig 2B) in almost all populations. Depending on the parameter configuration, either the low-activity fixed point has a sufficiently large basin of attraction for the simulated activity to remain near it, or fluctuations drive the network to the high-activity fixed point. To counter the shortcoming of vanishing infragranular firing rates, we define an additional parameter *κ* which increases the external drive onto 5E by a factor *κ* = *K*_ext,5E_/*K*_ext_ compared to the external drive of the other cell types. Since the rates in population 6E are even lower, we increase the external drive onto 6E linearly with *κ* such that *κ* = 1.15 results in *K*_6E,ext_/*K*_ext_ = 1.5. However, even a small increase in *κ* already drives the network into the undesired high-activity fixed point (Fig 2B). The stabilization procedure described by [37] uses mean-field theory to determine the population-averaged firing rates characterizing the fixed points of the system (cf. Eqs (1) and (2)). By linearizing the population dynamics around the fixed points, the technique identifies connectivity components that are most critical to the global stability of the fixed points and yields targeted modifications of the connectivity within the margins of uncertainty of the anatomical data. The resulting average relative change in total indegrees (summed over source populations) is 11.3%. This allows us to increase *κ* while retaining the global stability of the low-activity fixed point. In the following, we choose *κ* = 1.125, which gives *K*_6E,ext_/*K*_ext_ = 1.417, and *g* = 11, *ν*_ext_ = 10 spikes/s, yielding reasonable firing rates in populations 5E and 6E (Fig 2C) with sufficient global stability of the low-activity fixed point [37].

The stabilization renders the intrinsic connectivity of the areas more heterogeneous. Cortico-cortical connection densities similarly undergo small changes, but with a notable reduction in the mutual connectivity between areas 46 and FEF. For more details on the connectivity changes, see [37]. In total, the 4.13 million neurons are interconnected via 2.42 ⋅ 10^10^ synapses in the stabilized model.

### Area- and population-specific activity in the resting state

The network displays a reasonable ground state of activity with low spiking rates between 0.05 and 11 spikes/s (Fig 3). Inhibitory populations are generally more active than excitatory ones across layers and areas despite the identical intrinsic properties of the two cell types. This behavior, first found and discussed in detail in [36], is thus caused by the network connectivity which leads to a high excitation-inhibition ratio onto inhibitory cells. Spiking activity is asynchronous irregular across populations. Population activity fluctuates around its stationary point with small amplitude. Pairwise correlations are low throughout the network (Fig 3E). Excitatory neurons are less synchronized than inhibitory cells in the same layer, except for L4. Spiking irregularity is close to that of a Poisson process across areas and populations (Fig 3F). The only exception is population 6E, which features very low firing rates, so that the measure probably suffers from insufficient spiking data in single cells.

### Inter-area coupling leads to metastable regime

**Fig 4:**
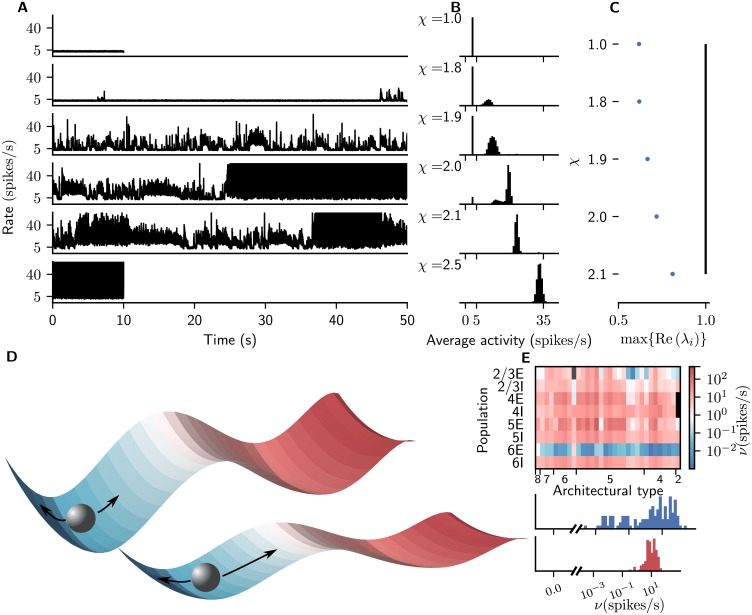
Emergence of metastable network dynamics. (A) Time course of spike rates averaged across all neurons in area V1 for increasing *χ* (graphs from top to bottom). Cortico-cortical synaptic weights onto inhibitory cells are additionally scaled by $\chi_{I}=2$ for *χ* > 1. (B) Histogram of analytically computed population firing rates resulting from 10^4^ random initial conditions of Eq (2) for same *χ* as in (A) averaged across all populations of the network. (C) Real part of maximal eigenvalue of the effective connectivity matrix (Eq (3)) for different values of *χ*. The values stay below 1 for *χ* ≤ 2.1, i.e., the network stays locally stable. The increasing proportion of initial conditions in panel B leading to the high-activity fixed point shows that at the same time, the global stability of the low-activity fixed point is reduced. (D) Sketch of the network phase space consisting of two stable fixed points and a submanifold with unstable fixed points separating the two basins of attraction (color indicates spike rate). The network activity (gray sphere) fluctuates around the low-activity fixed point (top). For increasing *χ* (bottom), the network approaches the separatrix, where the fluctuations become slow. If they are large enough, the network state can pass the separatrix and undergo a transition to the high-activity fixed point. (E) Population-resolved firing rates for *χ* = 1.9. Same display as in Fig 2.

To control interactions between areas, we scale cortico-cortical synaptic weights onto excitatory neurons by a factor $\chi =J_{\text{cc}}^{E}/J$ and provide balance by increasing the weights $J_{\text{cc}}^{I}$ onto inhibitory neurons by twice this factor, $J_{\text{cc}}^{I}=\chi_{I}\chi J=2\chi J$. For increasing *χ*, we observe growing fluctuations of the population spiking rates. At *χ* = 2 and beyond, the network enters a high-activity state at some time point in the simulation, where most populations spike at unrealistically high rates (Fig 4A). Predictions of mean-field theory show that for increasing *χ*, a growing proportion of initial conditions (in Eq (2)) result in states with increased activity (Fig 4B). We explain this behavior with the global phase space of the model. At any time, there are two stable attractors with basins of attraction divided by the separatrix, a hyperplane in the phase space that contains unstable fixed points. The low-activity fixed point remains locally stable for increasing *χ*, as determined by the maximal real part of the eigenvalues of the effective connectivity matrix which is below one for all configurations (Fig 4C). At the same time, its global stability, determined by the proportion of initial conditions leading the system to evolve to it, decreases (Fig 4B and 4D). The effect is that fluctuations around the stationary state, which are evident in a stochastic system, let the system approach the separatrix more closely. Close to the unstable fixed points, the dynamics of the system slow down, which causes the rate fluctuations to appear. From *χ* = 2, the system is likely to enter the high-activity state within a short amount of simulation time.

**Fig 5:**
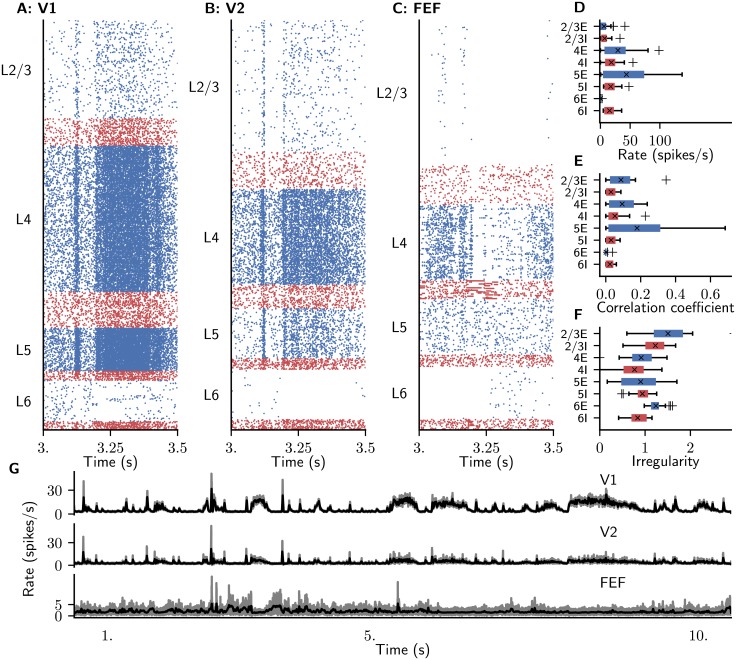
Resting state of the model with *χ* = 1.9. (A-C) Raster plot of spiking activity of 3% of the neurons in area V1 (A), V2 (B), and FEF (C). Blue: excitatory neurons, red: inhibitory neurons. (D-F) Spiking statistics across all 32 areas for the respective populations shown as area-averaged box plots. Crosses: medians, boxes: interquartile range (IQR), whiskers extend to the most extreme observations within 1.5×IQR beyond the IQR. (D) Population-averaged firing rates. (E) Average pairwise correlation coefficients of spiking activity. (F) Irregularity measured by revised local variation *LvR* [82] averaged across neurons. (G) Area-averaged firing rates, shown as raw binned spike histograms with 1 ms bin width (gray) and convolved histograms, with a Gaussian kernel (black) of optimal width [80].

In the following, we choose *χ* = 1.9 as the parameter configuration where slow fluctuations coincide with a sufficient global stability of the LA fixed point so that the system does not enter the HA fixed point during the simulation. The corresponding activity is irregular with plausible firing rates (Fig 5A–5C). Irregularly occurring population bursts of different lengths up to several seconds (Fig 5G) arise from the asynchronous baseline activity (Fig 5A–5C) and propagate across the network. The time scales of the population bursts arise from network interactions rather than directly reflecting axonal delays or membrane and synaptic time constants, which only cover a range of 10^0^ ∼ 10^1^ ms. The firing rates differ across areas and layers and are generally low in L2/3 and L6 and higher in L4 and L5, partly due to the cortico-cortical interactions (Fig 5D). The overall average firing rate is 14.6 spikes/s, with the inhibitory populations tending to have higher rates than the excitatory populations. However, the strong participation of L5E neurons in the cortico-cortical interaction bursts causes these to fire more rapidly than L5I neurons. Pairwise correlations are low throughout the network (Fig 3E). Unlike in the model without population bursts, excitatory neurons are more synchronized than inhibitory cells in the same layer, except for L6. Spiking irregularity is close to that of a Poisson process across areas and populations, with excitatory neurons tending to fire more irregularly than inhibitory cells (Fig 3F). Higher areas exhibit bursty spiking, as illustrated by the raster plot for area FEF (Fig 5C).

**Fig 6:**
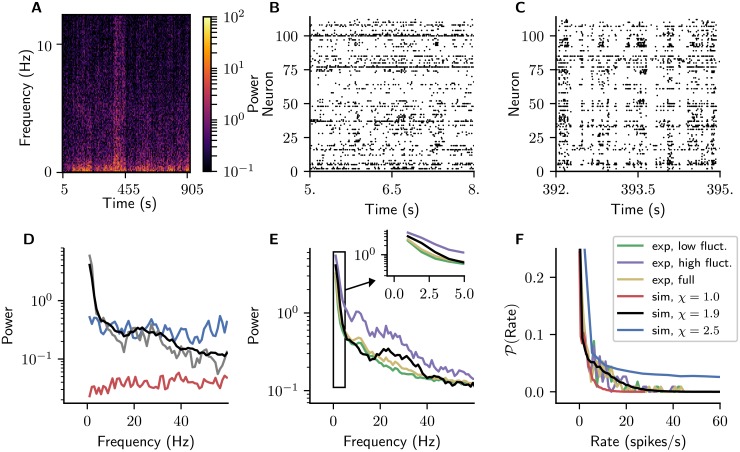
Comparison of the spiking dynamics with experimental data. Spike recordings from 140 neurons in primary visual cortex of macaque monkey [38, 104]. (A) Spectrogram of the spike histogram across all neurons. (B) Raster plot of spiking activity for the initial phase, *t* ∈ [5 s, 8 s]. (C) Raster plot of spiking activity in the later phase, *t* ∈ [392 s, 395 s]. Neurons are sorted according to depth of the recording electrodes with neurons closest to the surface of the brain at the top. (D) Power spectral density (PSD) of spike histograms for 140 neurons randomly sampled from all populations of V1 for three different networks simulated for *T* = 10 s with *χ* = 1(red), *χ* = 1.9 (gray) and *χ* = 2.5 (blue) and the power spectral density for *χ* = 1.9 for a simulation time of *T* = 100 s (black). (E) Comparison of simulated power spectral density with *χ* = 1.9 for 140 neurons randomly sampled from all populations of V1 (black) with experimental recording during low-fluctuation (green) and high-fluctuation (purple) phases and the full recording (yellow). Inset: enlargement for frequencies up to 5 Hz. All power spectra were computed using Welch’s method (see Material and methods). (F) Distribution of spike rates across single cells in experimental data (green, purple, yellow) and simulated spike trains across all layers and populations of V1.

We compare the simulated spiking activity with experimental data from [38], who recorded spiking activity in 140 neurons of macaque primary visual cortex in the spontaneous condition. The experimental activity shows activity phases differing in their low-frequency power (Fig 6A). In the early stage of the recording, the population activity exhibits only small fluctuations (Fig 6B), while in later stages, the population activity fluctuates on different time scales up to the order of a second (Fig 6C). We therefore split the recorded data into low-fluctuation and high-fluctuation phases (see Materials and methods for details), distinguished by their power at frequencies up to 40 Hz (Fig 6E). To compare simulated with recorded power spectra, we compute the spike rates of 140 cells in V1 distributed across populations in proportion to the population sizes (see Materials and methods for details). We compare three different simulations with low fluctuations (*χ* = 1, Fig 3), meta-stable dynamics (*χ* = 1.9) and unrealistically high activity (*χ* = 2.5) in Fig 6D. The power spectral densities (PSD) of the simulations with *χ* = 1, 2.5 are flat while for *χ* = 1.9 the PSD clearly reflects the slow oscillations in the spiking activity (cf. Fig 5). We compare the PSD of this simulation with the experimental results. Overall, the simulated activity in V1 to a good approximation reproduces both the spectrum from the entire recording period and that from the low-fluctuation phase, differing mainly in its increased power between 20 and 40 Hz. The sum of squared deviations (SSD) of the logarithmized spectrum from the logarithmized experimental spectrum for the entire recording period is SSD = 44 (*χ* = 1.9), compared to SSD = 793 for weak cortico-cortical synapses (*χ* = 1) and SSD = 2180 for strong cortico-cortical synapses (*χ* = 2.5), showing that this match is unique to the metastable case. For the entire frequency range, the metastable case (*χ* = 1.9) best matches the low-fluctuation phase (SSD = 42 vs. SSD = 89 for the high-fluctuation phase). At frequencies below 3 Hz, the power spectrum of the simulations closely matches that of the high-fluctuation phase (*χ* = 1.9: SSD = 3.2 vs. SSD = 6.8 for the low-fluctuation phase). The horizontal stripes in Fig 5I and 5J may to some extent be due to the mixing of spike trains from excitatory and inhibitory neurons, as the spike sorting does not distinguish between these. This interpretation is supported by the fact that the simulated activity across all layers and populations of V1 closely reproduces the broad distribution of spike rates across cells (Fig 5L). The model with weak cortico-cortical synapses has an overrepresentation of near-silent neurons, while that with strong cortico-cortical synapses overrepresents neurons with high firing rates, and both settings lead to flatter spectra than observed experimentally. Thus, the close match between simulated and experimental population spectra and firing rate distributions is specific to the metastable state.

### Structural and hierarchical directionality of spontaneous activity

**Fig 7:**
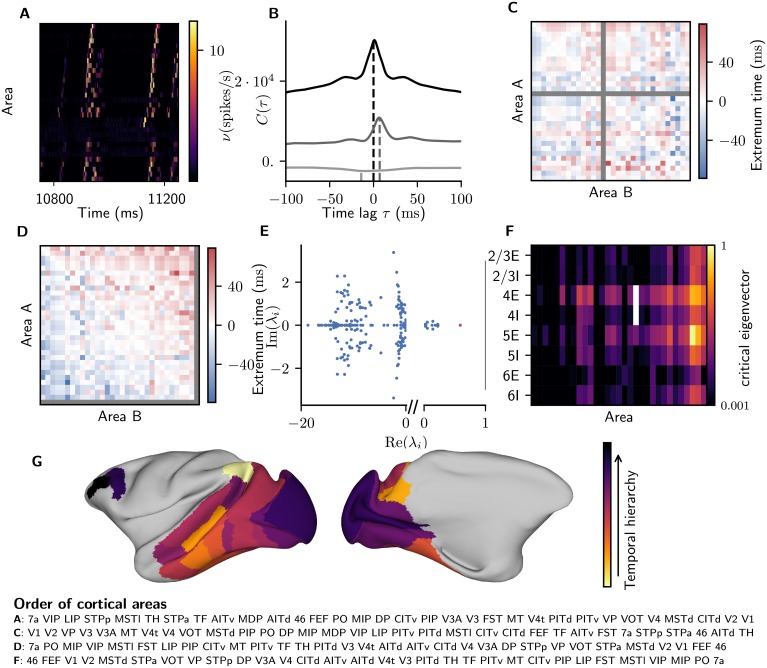
Temporal hierarchy. (A) Area-averaged firing rates (color code) for a sample period (horizontal), with areas (vertical) ordered according to the onset of increased activity. (B) Covariance functions of the area-averaged firing rates of V1 with areas V2 (gray) and FEF (light gray), and auto-covariance function of V1 (black). Dashed lines mark time lags detected by a wavelet smoothing algorithm (see Materials and methods). (C) Matrix of time lags of the correlation function for all pairs of areas. Area MDP is neglected (gray matrix elements) because it has only outgoing connections to but no incoming connections from other visual areas presently included in CoCoMac. Areas are ordered according to their architectural type. (D) Same matrix as in C, but with areas ordered according to the temporal hierarchy. (E) Eigenvalues of the effective connectivity matrix (see Materials and methods). Red, eigenvalue with maximal real part. (F) Absolute value of the corresponding critical eigenvector projected onto the populations of the model on logarithmic scale. Areas are ordered according to their position in the temporal hierarchy. (G) Lateral (left) and medial (right) view on the left hemisphere of an inflated macaque cortical surface showing the order in which areas are preferentially activated (color code). Created with the “view/map 3d surface” tool on https://scalablebrainatlas.incf.org.

To investigate inter-area propagation, we determine the temporal order of spiking (Fig 7A) based on the correlation between areas. We detect the location of the extremum of the correlation function for each pair of areas (Fig 7B) and collect the corresponding time lags in a matrix (Fig 7C). In analogy to structural hierarchies based on pairwise connection patterns [92, 93], we look for a temporal hierarchy that best reflects the order of activations for all pairs of areas (see Materials and methods). The result (Fig 7D) places parietal and temporal areas at the beginning and early visual as well as frontal areas at the end. The first and second halves of the time series yield qualitatively identical results. Fig 7D visualizes the consistency of the hierarchy with the pairwise lags: positive (negative) time lags are placed in the upper (lower) triangle of the reordered time lag matrix. To quantify the goodness of the hierarchy, we counted the pairs of areas for which it indicates the reverse order compared to that of the cross-correlation peaks. The number of such violations is 196 out of 496, well below the 230 ± 12(SD) violations obtained for 100 surrogate matrices, created by shuffling the entries of the original matrix while preserving its antisymmetric character. This indicates that the simulated temporal hierarchy reflects nonrandom patterns. The propagation is mostly in the feedback direction not only in terms of the structural hierarchy, but also spatially: activity starts in parietal regions, and spreads to the temporal and occipital lobes (Fig 7G). However, activity troughs in frontal areas follow peaks in occipital activity and thus appear last. This predominant feedback propagation occurs despite feedforward connections on average constituting a greater proportion of the connections onto the neurons in the network than feedback connections, indicating that the dynamical state to an important extent determines the effective strength of anatomical connections. In particular, the high firing rates of the excitatory populations in layer 5 compared to those in layer 2/3 enhance the influence of feedback compared to feedforward projections, as feedforward and feedback projections arise predominantly from the supragranular and infragranular layers, respectively. We analyze the eigenspectrum of the effective connectivity matrix (Fig 7E, cf. Fig 4) and find that the most critical eigenvector (whose eigenvalue has the largest real part, marked in red in Fig 7E) has the largest contributions in the areas at the bottom of the temporal hierarchy. To test whether the local structure of the areas, particularly the increased indegree in higher areas, alone predicts the relative instability in higher areas, we perform another test: We compute the maximum real part of the eigenvalues of the gain matrix in isolated areas with mean-field theory, where we replace inputs from other populations by Poisson input with a global rate of *ν*_ext_ = 10 Hz. This does not yield a systematic correlation with the position of the areas in the temporal hierarchy. We conclude that areas at the bottom of the temporal hierarchy are the most unstable areas in the network, i.e., fluctuations in these areas are less suppressed than in temporal and occipital areas, and that this is not a local effect but rather caused by the global network structure.

### Emerging interactions resemble experimental functional connectivity

**Fig 8:**
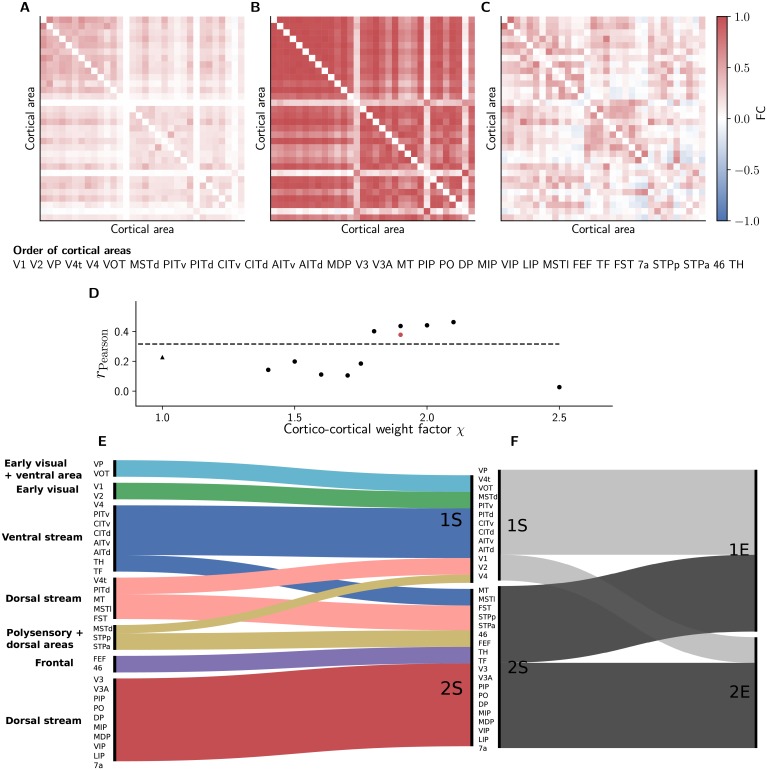
Inter-area interactions. (A) Simulated functional connectivity (FC) for *χ* = 1.9 measured by the zero-time-lag correlation coefficient of synaptic input currents. (B) Functional connectivity obtained by applying the Balloon model of [94] to the simulated synaptic input currents. (C) FC of macaque resting-state fMRI (see Materials and methods). The matrix elements are sorted according to simulated clusters determined with the Louvain algorithm [112] (see Materials and methods). (D) Pearson correlation coefficient of simulated FC vs. experimentally measured FC as a function of scaling factor *χ* for cortico-cortical synaptic weights with $\chi_{I}=2$ (dots) and $\chi_{I}=1$ (triangle, cf. Fig 3). Red dot, Pearson correlation coefficient between simulated FC based on BOLD signal and experimentally measured FC. Dashed line, Pearson correlation coefficient of structural connectivity vs. experimentally measured FC. Bottom row: Alluvial diagrams [113] showing (colors distinguish clusters) the differences in the clusters for the structural connectivity and the simulated FC (E), and simulated and experimentally measured FC (F). The clusters of the structural connectivity are extracted using the map equation method [98].

We compute the area-level functional connectivity (FC) based on the synaptic input current to each area, which has been shown to be more comparable to the BOLD fMRI than the spiking output [107]. The FC matrix exhibits a rich structure, similar to experimental resting-state fMRI (Fig 8A and 8C, see Materials and methods for details). In addition, we use the Balloon model of [94] to compute a BOLD signal from the area-averaged synaptic inputs (see Materials and methods for details). The resulting matrix displays a similar structure as the FC matrix based on synaptic input currents. Overall, the values tend to be more extreme (closer to +1 or −1). There are several possible reasons for this: Since our network model comprises the visual cortex only and does not consider neuromodulation, it is potentially in a more confined state than a larger, neuromodulated model of this type and may therefore be less noisy than real brains. Furthermore, the spatial convergence and divergence of cortico-cortical connections presumably also contribute to the variability while in the model all cortico-cortical connections emanate from and target 1 mm^2^ of spatially unresolved microcircuitry. Lastly, the experimental data are averaged over six monkeys and inter-individual variability decreases the average absolute FC values due to a spread between both negative and positive values. In particular, the positive and negative FC values considered separately are larger in individual monkeys than in the averaged matrix.

In the simulation, frontal areas 46 and FEF are more weakly coupled with the rest of the network compared to the experiment, but the anticorrelation with V1 as also found by [108] is captured by the model (the corresponding entries in Fig 8A are light blue, see also Figs 8B, 5A and 5C). Area MDP sends connections to, but does not receive connections from other areas according to CoCoMac, limiting its functional coupling to the network. The structural connectivity of our model shows higher correlation with the experimental FC (*r*_Pearson_ = 0.34) than the binary connectivity matrices from both a previous [109] and the most recent release of CoCoMac (*r*_Pearson_ = 0.20), indicating the importance of taking into account connection weights. For $\chi =\chi_{I}=1$, areas interact weakly, resulting in low correlation between simulation and experiment (Fig 8D). For increasing weight factor *χ* (with $\chi_{I}=2$), the correlation between simulation and experiment improves. For intermediate cortico-cortical connection strengths, the correlation of simulation vs. experiment exceeds that between the structural connectivity and experimental FC, both for FC based on synaptic currents (*r*_Pearson_ = 0.44) and simulated BOLD signal for *χ* = 1.9 (*r*_Pearson_ = 0.38, red dot in Fig 8D) indicating the enhanced explanatory power of the dynamical model. To compare the agreement of our simulated FC with the inter-individual variability between the six monkeys used in the experiments, we compute the average correlation across the monkeys to be 0.31. Comparing the simulated FC (based on synaptic currents) to the FC of individual monkeys yields an average correlation of *r*_Pearson_ = 0.39 ± 0.04, showing that the agreement between the simulated and experimental FC reaches the upper limit determined by the inter-individual variability. From *χ* = 2 on, the network is increasingly prone to transitions to the highly active state (cf. Fig 4), causing the correlation to decrease. Thus, the highest correlation occurs in a state in which the model exhibits metastable dynamics and slow rate fluctuations appear. Such dynamical slowing close to instability has previously been demonstrated in models of cortical resting-state dynamics where the individual areas were described by population rate equations [110] or small numbers of spiking neurons [111].

Louvain clustering [112], an algorithm optimizing the modularity of the weighted, undirected FC graph [96], yields two modules for both the simulated and the experimental data (Fig 8E). We denote the simulated modules by 1S, 2S and the experimental ones by 1E, 2E and compare these dynamical clusters with the community structure of the structural connectivity. In [35], we describe six clusters in the connectivity exposed using the map equation method [98]. Applying the same method to the modified connectivity matrix used for the simulations in the present work yields seven clusters that are similar to the clusters of the original connectivity, and reflect known functional groupings (Fig 8E). Three clusters contain dorsal stream areas and one large cluster gathers ventral stream areas. Furthermore, early visual areas and frontal areas each form separate communities. Ventral area VOT is grouped together with early visual area VP.

The modules exposed by simulation combine these structural clusters. Cluster 1S contains early visual along with ventral and three dorsal regions. Cluster 2S merges parahippocampal with dorsal but also frontal areas. The experimental module 2E comprises early visual areas, ventral area V4, and dorsal areas, while 1E consists of all other areas including also eight dorsal areas. This large degree of cross-over is most likely caused by an underestimation of the interactions between the areas in module 1E: ventral stream, frontal, and parahippocampal areas, and a subset of dorsal areas, since these are more strongly correlated in the experimental than in the simulated data.

In conclusion, our analysis, summarized in Fig 8, shows that the interactions between areas in the network resemble resting-state fMRI data and the agreement is highest when the network is in the metastable state at intermediate cortico-cortical connection strength.

### Population-specific cortico-cortical interactions

**Fig 9:**
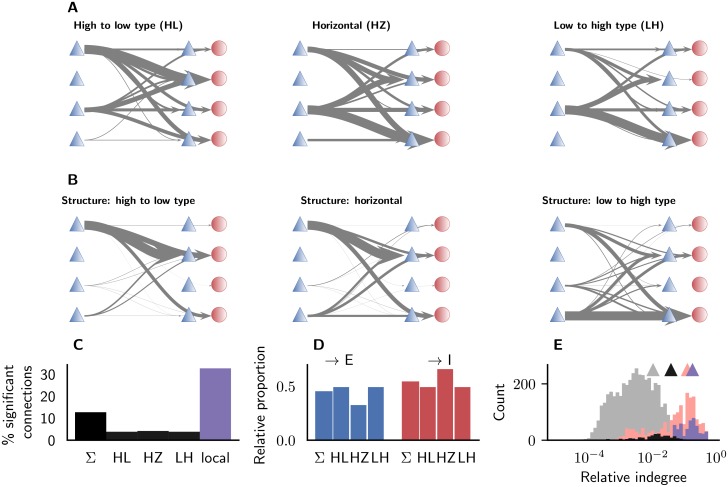
Laminar interactions. (A) Significant causal interactions between all pairs of areas categorized according to the difference between their architectural types. Arrow thickness indicates the occurrence of significant interactions (*p* < 0.05, Levene’s test) between the particular populations (cell types and layers displayed as in Fig 1). (B) Population-specific patterns of shortest paths between all pairs of areas. (C) Proportion of significant connections for the three categories and local connections. (D) Proportion of significant impact onto excitatory and inhibitory populations, respectively, for the three different categories. *Σ* indicates the respective value over all connections. (E) Histogram of relative indegrees. Bars show data for all (light gray) and significant (black) cortico-cortical connections; and for all (pink) and significant (purple) local connections. The triangles indicate the mean values of the four distributions.

We investigate the laminar patterns of cortico-cortical interactions by computing conditional Granger causality on pairs of connected populations in different areas. Testing for significance (*p* < 0.05) with Levene’s test [100] yields the pairs of causally influencing populations. We distinguish area pairs into three different categories based on the relation of their structural types. These categories approximately agree with the commonly used terminology of feedforward (∼high-to-low-type), lateral (∼horizontal) and feedback (∼low-to-high-type) directions. We observe systematic differences between the three different categories (Fig 9A). High-to-low-type interactions preferentially originate in the supragranular layer and target layer 4, while low-to-high-type interactions are controlled by population 5E and target the infragranular layers. Horizontal interactions share features of both other types. We compare the patterns of dynamical interactions with patterns of shortest paths in the structural connectivity between areas (Fig 9B). High-to-low-type interactions resemble the shortest paths, but the other two types show clear differences to structural paths. Dynamical interactions in the horizontal direction are more similar to low-to-high-type interactions while horizontal shortest paths resemble high-to-low-type paths. The low-to-high-type interactions are dominated by population 5E, while the shortest paths suggest a stronger influence of population 6E. The shortest paths in the structural connectivity differ from the results of [35] because we here consider the modified connectivity after stabilization by the method of [37] as well as synaptic weights with an inter-area scaling factor of *χ* = 1.9 and respect the population-specific firing rates as opposed to [35], where all populations are assumed to have equal activity levels. The detected shortest paths are nonetheless similar to the ones presented in Figure 8 of [35]. The most obvious difference is that paths onto inhibitory populations are significant in the simulated network (Fig 9D), most likely due to the increased synaptic weight of corticocortical projections onto inhibitory populations. Furthermore, in low-to-high-type connections, paths onto L6 are more relevant.

Overall, the number of significant connections is low, with approximately 4% of the cortico-cortical connections leading to significant causal interactions (Fig 9C). Locally, one third of the connections carries causal interactions between the populations of an area. This observation reflects the high degree of local connectivity in cortex. The indegrees of connections with significant interactions are higher compared to all connections, for both cortico-cortical and local connections (Fig 9E). Still, there is no strict dependence of causal interactions on high indegrees, since there are weak but causal connections as well as strong, non-causal connections. One reason is that the activity level of the projecting population plays a major role. The connectivity structure alone predicts for instance that 6E plays a dominant role in low-to-high-type projections, but these connections are actually not significant in the simulations. This is caused by the low activity level of 6E rendering these populations only influential in local interactions. Also the activity of the target populations modulates the effective interactions by determining the susceptibility to inputs due to the nonlinearity of the firing rate response curve [114, 115]. In this sense, network simulations are more powerful than studies merely considering structural connectivity because they add these dynamical aspects to the cortico-cortical interactions. In conclusion, causal interactions in the network, summarized in Fig 9, follow laminar patterns that depend on the relative architectural differentiation of areas. This dependence results from a combination of structural connectivity differences and dynamical states of source and target populations.

## Discussion

We present simulations of a multi-scale spiking network model of macaque visual cortex relating cortical connectivity to its dynamics. The connectivity [35] is refined by a mean-field-based stabilization procedure [37] incorporating fundamental activity constraints of non-vanishing, non-saturating spiking rates. The network produces a state with single-cell spiking statistics close to those from recordings in macaque V1 along with large-scale interactions resembling inter-area correlation patterns in macaque fMRI. The model predicts that cortex operates in a metastable regime, and exposes layer-specific, hierarchically organized channels mediating inter-area interactions.

Population firing rates are layer-specific, inhibitory rates generally exceeding excitatory ones, in line with experiments [4, 116, 117]. Since excitatory and inhibitory neurons are equally parametrized and excitatory neurons receive equal or stronger external stimulation compared to inhibitory ones, we conclude that the connectivity causes these differences. The contribution of faster intrinsic dynamics of inhibitory neurons [118, 119] merits future investigation. The mean firing rate of the network is not far above the mean spontaneous rate in V1 of alert monkeys [2]. However, common recording methods may miss (near-)silent neurons that would lower overall rates [120]. Future work can address this potential discrepancy.

With sufficiently strong cortico-cortical connections, fluctuations let the system approach an instability where the dynamics drastically slow down [110, 111, 121, 122]. In this metastable regime, the network closely reproduces the spike rate distribution across V1 neurons in lightly anesthetized macaque [38]. This state features variable-length population bursts mediating inter-area interactions, resembling cortical synchronized fluctuations in both spontaneous and stimulus conditions observed in mouse [123], rat [124, 125], and macaque [7, 104]. Overall, our simulations to a good approximation match the power spectrum of [38], matching periods of higher synchrony at low frequencies. Simultaneous recordings of spontaneous activity in V1 and eye movements of a monkey sitting in darkness suggest that higher synchrony reflects drowsiness [126]. The low-frequency fluctuations may represent irregular Up-Down fluctuations produced by a bistable network with transitions between Up and Down states driven by external input to each area rather than arising locally from an adaptation mechanism, consistent with a recent experimental and modeling study [125]. Further work could distinguish such network effects from other sources of low-frequency fluctuations including NMDA and GABA_B_ transmission, neuromodulation, and adaptation.

The pattern of simulated interactions resembles fMRI resting-state activity from the anesthetized macaque. The functional connectivity (FC) based directly on the synaptic inputs provides similar predictions to that computed using the Balloon model, indicating that the low-frequency fluctuations present already at the level of the spiking activity drive these interactions. The agreement between simulation and experiment peaks at intermediate coupling strength in the metastable regime, potentially related to the slight subcriticality of the resting brain [127]. By combining these large-scale dynamics with plausible single-cell spiking statistics and layer-specific communication, our study extends earlier models exhibiting similar behavior with simpler local circuits [18, 128]. We compute a BOLD signal based on the Balloon model of [94] from the simulated spiking activity and find that the resulting functional connectivity resembles the experimental data, but that the absolute values of the functional connectivity are larger than for the experiment and the synaptic input currents of the model. The model may underestimate the level of noise and thereby overestimate the functional connectivity due to the restriction to visual cortex and the lack of neuromodulatory effects, as well as the lack of convergence and divergence in cortico-cortical connections beyond the 1 mm^2^ scale. On the other hand, the experimental functional connectivity may be underestimated due to averaging of the experimental data across monkeys, which leads to a decrease in the absolute values owing to inter-individual variability.

There is an ongoing debate in the literature regarding the extent to which fluctuations of functional connectivity during the resting state (e.g. [129, 130]) are significant. Our simulations do not yield slow alternations between the clusters in our FC matrix. There are various possible reasons: experimentally observed FC dynamics in the resting state may be largely due to sampling variability and head motion [131]; the limited duration over which we can simulate and save the corresponding spiking data, which becomes gigabytes or even terabytes in size, is still too short to enable FC dynamics to be observed [132]; or FC dynamics may depend on variations in cognitive state [133] or vigilance [130]. Because we do not simulate the rest of the brain nor neuromodulation, such variations in cognitive and vigilance states are not captured.

The model construction starts with the binary connectivity, defines the weighted structural connectivity based on connection densities, and finally yields the functional connectivity from simulations. Comparing with the experimentally observed functional connectivity, we find that each level (binary structure → weighted structure → dynamical simulation) adds explanatory power. In relation to fMRI functional connectivity, the role of anesthesia is worth investigating, as anesthetics influence BOLD responses in complex ways, although low doses as used in the experiment described here may have only mild effects, as shown in rat [134] and marmoset [135].

The model predicts that population bursts propagate stably across multiple areas, predominantly in the feedback direction, because parietal areas are more unstable than temporal and occipital areas, as revealed by linear stability analysis. This occurs despite higher relative indegrees of feedforward connections, indicating the importance of the dynamical state for the effective influence of the structural connectivity. Specifically, the comparatively high firing rate of the layer 5 excitatory neurons enhances the influence of feedback compared to feedforward connections, which mainly originate in supragranular layers. The systematic activation of parietal before occipital areas in the model parallels human EEG findings on information flow during visual imagery [136] and top-down slow-wave propagation during sleep in humans [89, 90] and mice [91]. Our method for determining the order of activations resembles one recently applied to fMRI recordings [10]. It could be extended to distinguish between excitatory and inhibitory interactions like those we observe between V1 and frontal areas or to identify multiple ‘lag threads’ in the network [137].

Granger causality analysis of cortico-cortical interactions reveals layer- and population-specific communication channels that depend on the difference in the architectural types of the areas. Low architectural types correspond to low neuron density and a thin or absent layer 4 (more limbic areas); high architectural types correspond to high neuron density and a pronounced layer 4 (more sensory areas) [50, 53, 54, 138]. In terms of visual processing hierarchies, areas of high architectural type are found at the bottom of such hierarchies while areas of low type constitute the top level of visual hierarchies. Interactions from high to low types, corresponding to feedforward communication, originate in layer 2/3 and target layer 4, while interactions from high to low types, associated with feedback communication, are predominantly mediated by source neurons in layer 5 and target neurons in layers 6 and 4. Thus, layer 5 neurons are the dominant source of feedback interactions, in contrast with an equal division between layers 5 and 6 in terms of anatomical connectivity [35]. This distinction is due to the higher activity in layer 5 than in layer 6 which enters the Granger causality analysis on both source and target side, whereas it influences only the target side in the path analysis of the structural connectivity via the gain of the receiving population. These findings differ from existing theories about predictive coding in cortical microcircuits [139, 140] insofar that feedback signals preferentially reach granular and infragranular neurons rather than supragranular neurons. This suggests a more prominent role of layer 4 because in addition to feedforward signals and intrinsically produced feedback predictions (via the local L2/3→L5/6→L4 pathway), statistical mapping of synapses to target neurons according to dendritic length [51] provides it with direct feedback predictions from higher areas via apical dendrites in upper layers. Incorporating a dual counterstream organization of feedforward and feedback connections [66, 141] would allow a more refined analysis of these laminar interactions.

Comparing these results with the network structure shows that substantial effective communication arises over a range of connection strengths due to the influence of the dynamical state of the populations, but that the weakest connections do not contribute significantly to communication. Our insights open up a new perspective on the significance of weak ties [142], since our results suggest that they can gain relevance if they link highly active populations but their influence should not be overestimated by binarizing the connectivity. Incorporating local structure beyond population-specific connectivity of point neurons would enable studying how this could strengthen the influence of weak connections [143, 144]. Our analysis further stresses the dominance of local communication in cortical dynamics.

In the model, cortico-cortical indegrees are higher onto excitatory than onto inhibitory neurons, but stronger synapses onto inhibitory than onto excitatory neurons are required to maintain stability. This stabilization mechanism is similar to stabilization by inhibition locally in V1 [145], but uses a different way to balance excitation and inhibition. Locally, the activity of inhibitory neurons increases in proportion to the activity of excitatory neurons [6] via a feedback mechanism [146–149]. In the corticocortical interactions in our model, the activity of the excitatory and inhibitory neurons is increased proportionally by a common input. In principle, other features could support stable inter-area activity propagation, such as spike-frequency adaptation of excitatory neurons [150, 151], short-term synaptic plasticity [152–154], more detailed local balance [155], and fine-tuned excitatory and inhibitory pathways [156–162]. However, optogenetic photostimulation experiments in mice provide some support for our prediction: inter-area EPSCs onto parvalbumin-expressing (PV) interneurons were found to be stronger than onto pyramidal neurons in mouse visual cortex, and mean EPSCs per pixel were larger onto PV interneurons at least for feedforward connections [163]. This outbalancing of excitation by inhibition resembles the “handshake” mechanism in the microcircuit model of [36] where interlaminar projections provide stability by their inhibitory net effect. In the model, this stabilization is reflected in the slightly larger proportion of significant interactions onto inhibitory than onto excitatory populations. In contrast, [20] model feedback connections onto layer 5/6 as net excitatory and onto layer 2/3 as net inhibitory, to reflect the hypothesis that the latter convey predictions suppressing feedforward activation. However, feedback connections may be facilitatory for stimuli within the classical receptive field and suppressive outside the receptive field [139, 164–166]. Such details in feedback processing can be integrated and studied in future model versions.

Random connectivity below the population level is unlikely to suffice for the brain to perform its computations. Including higher-order structures such as neuron-level motifs [167] and patchy connections would make the connectivity more realistic and enable the network to support specific activity patterns between groups of neurons inside a population [168], such as synfire chains [169]. For instance, reciprocal connections and multiple synaptic contacts between neurons are overrepresented in cortex compared to random networks [156, 161, 162], and the existence and strength of a synaptic connection between two neurons correlates with the existence of connections with other neurons [156, 159]. Such cell-specific connectivity is likely to influence firing rate distributions, correlations, and propagation of activity; and theoretical studies have shown that they influence the computational capacity of cortical circuits [15, 170]. To determine the large-scale connectivity, we included only connections from CoCoMac and [30]. CoCoMac alone contains information on approx. 78% of all pairs of cortical areas in the scheme of [45] and approx. 66% of all pairs of visual areas. Including the data of [30], the existence or absence of a connection has been established for 76% of pairs of visual areas. It would be interesting to study the influence of additional connections predicted from graph-theoretical analyses [171]. Our model distinguishes only excitatory and inhibitory cells while cortical networks consist of many different cell types [162, 172]. In particular, integrating different types of inhibitory cells and their detailed projection patterns [173–177] would enrich the model dynamics [178, 179]. Furthermore, going beyond the simple single-neuron dynamics used in this study would enable one to study effects of intrinsic neuron dynamics, such as active calcium conductances in dendrites [180, 181], on the network state.

For tractability, the model represents each area as a 1 mm^2^ patch of cortex. True area sizes vary from ~3 million cells in TH to ∼300 million cells in V1 for a total of ∼8 ⋅ 10^8^ neurons per hemisphere of macaque visual cortex, a model size that with recent advances in simulation technology [28] already fits on the most powerful supercomputers available. Approaching this size would reduce distortions imposed by downscaling [34] and enable a more realistic representation of synaptic convergence.

Overall, our model elucidates multi-scale relationships between cortical structure and dynamics, and can serve as a platform for the iterative integration of new experimental data, the creation of hypotheses, and the development of functional models of cortex.
